# Quantity and Distribution of Eosinophils in Esophageal Specimens of Adults: An Iranian Population-Based Study

**DOI:** 10.30699/IJP.2021.520951.2546

**Published:** 2022

**Authors:** Elena Jamali, Behrang Kazeminezhad, Mahsa Ahadi, Afshin Moradi, Hamideh Khabbazi

**Affiliations:** 1 Department of Pathology, School of Medicine, Loghman Hakim Hospital, Shahid Beheshti University of Medical Sciences, Tehran, Iran; 2 Department of Pathology, School of Medicine, Shohada-e-Tajrish Hospital, Tehran, Iran

**Keywords:** Biopsy, Eosinophils, Esophagus, Resection

## Abstract

**Background & Objective:**

Eosinophils are normally found in different parts of the gastrointestinal tract and with less prevalence in the esophagus. Eosinophilic infiltration is increased as part of inflammatory reactions in various diseases. The aim of this study was to determine the count and distribution of eosinophils in esophageal specimens obtained for different causes.

**Methods:**

Endoscopy and pathology reports of esophageal specimens in Shahid Beheshti University related hospitals, Tehran, Iran, were extracted from 2016 to 2019. The prevalence of gastroesophageal reflux disease (GERD), malignancy, eosinophilic esophagitis, and asymptomatic patients were determined as the percentages of total resection and biopsy specimens. Each group was calculated and randomly selected according to the inclusion criteria. All data were analyzed statistically using SPSS software.

**Results:**

A total of 258 biopsy and resection specimens were evaluated in this study. Fourty three cases (16.7%) diagnosed as normal esophageal mucosa , 42 cases (16.3%) as non-specific esophagitis, 155 cases (60.1%) diagnosed as gastroesophageal reflux disease, 4 cases (1.6%) showed malignancy and other diagnoses were recorded for 14 cases (5.4%). The numbers of eosinophils in the epithelium and lamina propria in the normal group were 0.1±0.5 and 2.08±2.33, respectively. The eosinophil count in different groups and its relation to different histopathologic findings were diverse.

**Conclusion:**

The number of eosinophils within the lamina propria was significantly higher than those found within other layers. . The highest mean eosinophil count was observed in the epithelium and the lamina propria of cases diagnosed as GERD.

## Introduction

Eosinophils act as effective agents in defending the body against foreign factors as well as in tissue regeneration (1). Eosinophils are usually found in different parts of the gastrointestinal (GI) tract and are less commonly met in the esophagus's superficial epithelium and lamina propria. On the other hand, the obvious eosinophilic infiltration in the epithelium or eosinophilic degranulation is always abnormal. The presence of the eosinophils in the GI system is also affected by the environmental factors such as diet and geographic area. There is a significant increase in eosinophil count in the inflammatory conditions. The esophageal eosinophils are mainly the sign of chronic mucosal damage due to gastroesophageal reflux disease (GERD), although similar findings can be seen in eosinophilic esophagitis (EOE). A small number of eosinophils in the distal part of the esophagus without evidence of mucosal damage is insufficient to making a diagnosis of esophagitis. An increase in the number of eosinophils in the esophageal mucosa is also commonly associated with drugs and less commonly with infectious esophagitis. In all these conditions, eosinophils are seen admixed with variable numbers of lymphocytes and neutrophils. However, the differential diagnosis of pure eosinophilic infiltration is somewhat limited to hypersensitivity reactions and some infections (2). For decades, eosinophils were thought to be involved in only two mechanisms of fighting parasitic infections and allergic conditions, but recent studies showed that eosinophils might be involved in controlling inflammation, maintaining the epithelial barrier, participating in tissue remodeling, and immune system response (3-6). Previous mice studies have shown that eosinophils are present in the digestive system during infancy. This happens shortly after the GI tract microbiota is formed (7). In normal and disease-free regions, the numbers of the eosinophils increase orderly from esophagus to stomach, small intestine, and colon. In fact, from proximal to distal of the GI tract, the eosinophil count increases, and the maximum eosinophil count is seen in the cecum (8-10). Also, elevated eosinophil count is seen in some gastrointestinal diseases such as inflammatory bowel disease (IBD) and colorectal cancer (CRC).

Previous studies have also shown that diseases such as EOE are associated with elevated eosinophil count in different layers (1, 11-13). Evaluation of the mucosal eosinophilia plays an important role in interpreting endoscopic biopsies; For example, high eosinophil density can be the sign of allergic diseases. Despite the increasing prevalence of diseases associated with increa-sed eosinophil count in the GI tract, there is finite infor-mation about the normal eosinophil limits, abnormal increase, and pattern of eosinophil distribution in different layers (9, 10, 14). Therefore, the present study aimed to determine the eosinophil count and distribution in esophagectomy and biopsy specimens obtained from the hospitals of Shahid Beheshti University of Medical Sciences from 2016 to 2019.

## Material and Methods

The present diagnostic study was performed on 258 esophageal specimens. Endoscopic and pathological reports of esophageal biopsy and resection specimens were extracted from 2016 to 2018 by referring to the archives of the Pathology Departments of the hospitals of Shahid Beheshti University of Medical Sciences. According to the pathology report, a total of 258 available cases were classified in different diagnostic groups, including normal group, non-specific esophag-itis, GERD, esophageal malignancy, and miscellaneous diagnoses. The slides of each specimen were extracted and reviewed. The eosinophils in different layers of specimens were counted and other histopathological findings were recorded for each case based on the questionnaire.

**Statistical Analysis**

The collected data were analyzed with SPSS software (ver. 24). Also, the specialized statistical tests such as ANOVA, Kolmogorov–Smirnov test, variance, two-sample independent t-test, Pearson correlation test, and chi-square were performed for the categorical variables. P-value<0.05 was considered as the significant level in all tests**.**

## Results

The present study was carried out on 258 adult cases with a mean age of 50.44±16.70 years. The frequencies of females and males were 154 (59.7%) and 104 cases (40.3%), respectively. Based on the histopathological findings, the cases were distributed in different diagnostic groups as follow: normal (n=43, 16.7%), non-specific esophagitis (n=42, 16.3%), GERD (n=155, 60.1%), malignancy (n=4, 1.6%), and miscellaneous diagnoses (n=14, 5.4%).

Table 1 shows the eosinophil counts through different esophageal layers in different diagnostic groups. The ANOVA test showed that the average number of eosinophils in the epithelial and lamina propria was significantly different among diagnostic groups (*P*<0.01). The number of eosinophils in the lamina propria was significantly higher than those found in other layers in all groups.

**Table 1:** Eosinophil count in different esophageal layers based on different diagnoses

Diagnostic group		Epithelium	Lamina propria	Submucosa	Muscularis propria	Adventitia
Normal	Mean	0.10	2.08			
	N	40	25			
Non-specific esophagitis	Mean	0.67	4.42	2.75	3.00	**3.50**
	N	42	31	4	4	**4**
Gastroesophageal reflux disease	Mean	1.81	8.09			
	N	151	138			
Malignancy	Mean	2.00	3.50	4.67	1.00	**3.00**
	N	4	4	3	3	**3**
Other diagnoses	Mean	4.93	19.8	7.00	3.33	**1.67**
	N	14	10	3	3	**3**
P-value		**<0.01**	**<0.01**			

Evaluation of the average number of eosinophils, neutrophils, and lymphoplasma cells (LPCs) in different esophageal layers showed the frequency distribution of eosinophils, neutrophils, and LPCs at 0.99±0.06, 2.32±12.79, and 17.22±14.32 in the epithelial layer; 7.26±12.01, 3.67±16.11, and 82.53±64.29 in the LP; 4.60±6.64, 1.50±1.77, and 58.50±36.20 in the submucosa; 2.50±2.41, 3.20±5.47, and 53.70±29.78 in the muscularis propria; and 2.80±3.15, 5.40±6.68, and 46.60±33.50 in the adventitia, respectively.

Pearson correlation test was used to investigate the association between the average number of eosinophils and neutrophils in different esophageal layers (Table 2). The results showed a significant positive association between the number of eosinophils and neutrophils in the epithelial layer and the LP (*P*<0.001). There was no significant positive association between the number of eosinophils and neutrophils in the submucosa, muscularis propria, and adventitia, which affected the small number of specimens bearing these layers.

**Table 2:** Correlation of eosinophils and neutrophils in different esophageal layers

Group			Number of the eosinophils	Number of the neutrophils
Epithelium	Number of the eosinophils	Correlation Coefficient	1.000	0.437**
		P-value		**0.000**
		N	252	**252**
Lamina propria	Number of the eosinophils	Correlation Coefficient	1.000	0.283**
		P-value		**0.000**
		N	209	**206**
Submucosa	Number of the eosinophils	Correlation Coefficient	1.000	**0.441**
		P-value		**0.202**
		N	10	**10**
Muscularis propria	Number of the eosinophils	Correlation Coefficient	1.000	**0.230**
		P-value		**0.523**
		N	10	**10**
Adventitia	Number of the eosinophils	Correlation Coefficient	1.000	**0.631**
		P-value		**0.050**
**N**	**10**	**10**

**. Correlation is significant at the 0.05 level (2-tailed).

Pearson correlation test was used to investigate the association between the numbers of eosinophils and LPCs in different esophageal layers (Table 3). The findings showed a significant positive association between the number of eosinophils and LPCs in the LP (*P*<0.001). However, no significant positive association was found between the number of eosinophils and LPC in the epithelial, muscularis propria, submucosa, and adventitia layers.

In this study, the average numbers of eosinophils, neutrophils, and LPC in the total esophageal layers were 7.75±14.70, 5.63±20.19 and 90.10±82.27, respectively.

A two-sample independent t-test showed that the difference between the average number of eosinophils in epithelial and lamina propria layers in men and women was not statistically significant (*P*=0.88>0.05 and 0.60>0.05, respectively).

**Fig. 1:**
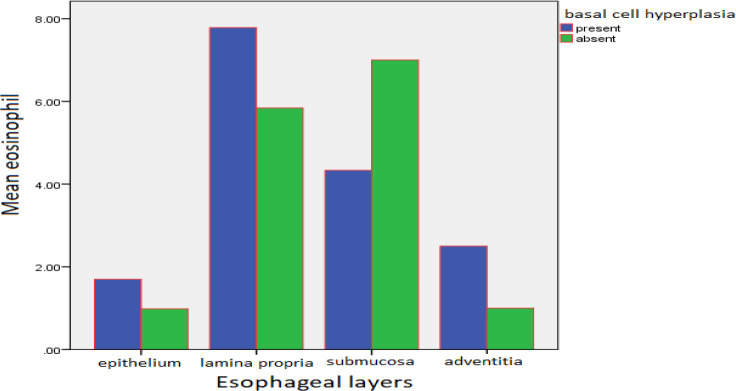
The average number of eosinophils in the presence and absence of basal cell hyperplasia in different layers

Basal cell hyperplasia (BCH) was seen in 9 cases (3.5%) of the normal diagnostic group, 23 cases (8.9%) of the non-specific esophagitis group, 141 cases (54.9%) of the GERD group, 3 cases (1.2%) of the malignancy group, and 10 cases (3.9%) of the miscellaneous diagnostic group (Figure 1).

There was no statistically significant difference in the average numbers of eosinophils in the epithelium at different degrees of BCH (*P*=0.14>0.05). However, in lamina propria, the difference in the average number of eosinophils was proportionate to different degrees of BCH (*P*=0.02<0.05).

**Table 3:** Correlation between the number of eosinophils and LPCs in different esophageal layers

Group			Number of the eosinophils	**Number of the lymphoplasma cells**
Epithelium	Number of the eosinophils	Correlation Coefficient	1.000	**-0.015**
		P-value		**0.818**
		N	252	**251**
Lamina propria	Number of the eosinophils	Correlation Coefficient	1.000	0.330**
		P-value		**0.000**
		N	209	**209**
Submucosa	Number of the eosinophils	Correlation Coefficient	1.000	**0.178**
		P-value		**0.623**
		N	10	**10**
Muscularis propria	Number of the eosinophils	Correlation Coefficient	1.000	**0.448**
		P-value		**0.194**
		N	10	**10**
Adventitia	Number of the eosinophils	Correlation Coefficient	1.000	0.667*
		P-value		**0.035**
**N**	**10**	**10**

*. Correlation is significant at the 0.05 level (2-tailed).

Similarly, there was a statistically significant difference in the average eosinophil numbers in lamina propria in the presence of elongation of the lamina propria papillae compared to the average eosinophils in the epithelium (*P*=0.04<0.05 and 0.52>0.005, respectively).

**Fig. 2:**
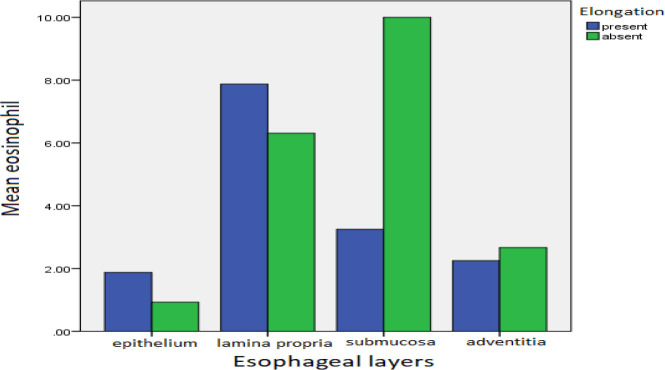
The average number of eosinophils in the presence and absence of elongation of the lamina propria papillae in different layers

In order to determine the average number of eosinophils in the epithelium and lamina propria in people with chronic inflammation, the ANOVA test showed a significant increase in the eosinophil infiltrate in proportion to the chronic inflammation intensity (*P*<0.05 in both layers).

ANOVA test also showed a significant increase in the average number of eosinophil infiltrate in the epithelium and lamina propria layers in proportion to acute inflammation intensity (*P*<0.01 in both layers).

In the specimens with the presence of distended pale squamous balloon cells, the average number of eosinophils in the epithelium and lamina propria layers was not statistically significant (*P*=0.19>0.05 and 0.22>0.05, respectively).

In the cases with the presence of intercellular edema, there was a statistically significant higher mean eosinophil number in epithelium and lamina propria layers (*P*<0.001 in both layers) compared to the absence of it (Tables 4 and 5).

**Table 4:** Frequency distribution of the average number of eosinophils in the epithelium in the presence of intercellular edema

Intercellular edema	N	Mean	95% Confidence Interval for Mean	
			Lower Bound	Upper Bound
Negative	28	0.357	0.073	**0.640**
Mild	157	0.592	0.321	**0.863**
Moderate	53	1.584	0.896	**2.273**
Severe	19	10.421	0.933	**19.908**
Total	**257**	**1.498**	**0.751**	**2.244**

**Table 5:** Frequency distribution of average number eosinophils in the lamina propria layer in the presence of intercellular edema

Intercellular edema	N	Mean	95% Confidence Interval for Mean	
			Lower Bound	Upper Bound
Negative	20	3.650	1.680	**5.619**
Mild	125	4.936	3.783	**6.088**
Moderate	47	10.383	5.189	**15.577**
Severe	17	20.000	10.128	**29.871**
Total	**209**	**7.263**	**5.624**	**8.901**

No statistically significant difference was observed in the average number of eosinophils in the epithelium and lamina propria in terms of the prese-nce/absence of surface erosion.

The two-sample independent t-test showed a statistically significant difference in the mean eosinophil number in the presence of fibrosis in lamina propria (*P*=0.002) (Table 6).

**Table 6:** Comparison of the average number of eosinophils in the lamina propria layer in the presence of fibrosis

Increased lamina propria fibrosis		N	Mean	Std. Deviation	Std. Error Mean
Number of the eosinophils epithelium	Present	74	3.351	10.814	**1.257**
	Absent	183	0.748	1.751	**0.129**
P-value	**0.002**				

## Discussion

The present study was carried out on 258 cases, of which 154 (59.7%) were women and 104 (40.3%) were men. One of the main objectives of the current study was to determine the mean number of eosinophils in different layers of the esophagus, especially in normal individuals, so that we can introduce a normal limit in the Iranian population to avoid overestimation of eosinophilia. The average numbers of eosinophils were 0.10±0.50 and 2.08±2.33 in the epithelium and LP layers, respectively. The statistical analysis showed a significantly higher average eosinophil count in the LP layer than the epithelial layer.

Matsushia et al. carried out a study on 132 normal Japanese populations to investigate the effect of race on the number of eosinophils in the GI tract and compared it with American and Caucasian races. They found that the number of mucosal eosinophils was clearly higher in all parts of the GI tract of the Asian race than Caucasian and American races. They also stated that the number of mucosal eosinophils in esophageal specimens was 0.07±0.43 (6).

However, in a study on gastrointestinal specimen biopsies in atopic and non-atopic individuals by DeBrosse, the number of eosinophils reported as 0.03±0.10 eosinophils per high-power field (hpf), and the highest eosinophil number was 1 eosinophil/hpf. They found a significant difference between the atopic and normal people in terms of eosinophils number in the biopsy specimens. In other words, the number of eosinophils in the biopsy specimens of atopic individuals was significantly different from that of non-atopic individuals (3).

In our study, the number of eosinophils in different esophageal layers was also assessed in various diagnostic groups. The highest eosinophil levels were found in the epithelium (average of 1.81±7.28) and lamina propria (average of 8.09±12.65) of GERD group, followed by non-specific esophagitis group with the average eosinophil numbers of 0.67±2.10 in the epithelium and 4.42±6.98 in the lamina propria that were clearly higher than normal group (epitheli-um=0.10±0.50 and the lamina propria=2.08±2.33). 

It was impossible to compare the malignancy and miscellaneous groups due to the small number of cases in these groups. The mean eosinophil count in the epithelial and lamina propria layers of eosinophilic esophagitis was not assessable in our study due to few cases in this category; however, there are defined criteria and eosinophil count cutoff for the eosinophilic esophagitis in the textbooks.

There was no significant difference between males and females in terms of the average number of eosinophils in the epithelial and lamina propria esophageal layers.

In addition, we evaluated the relationship between the eosinophil count and other histopathological factors and the results showed a significant positive association between the number of eosinophils and the number of neutrophils in the biopsy specimens, the number of LPCs in the lamina propria layer, degrees of basal cell hyperplasia, the presence of elongation of the lamina propria papillae, the presence of intercellular edema and fibrosis in lamina propria, as well as harmonized eosinophil count with intensities of acute and chronic inflammations. 

There was no significant statistical relationship between the number of eosinophils and LPCs in the epithelial layer, the number of eosinophils, the presence of distended pale squamous balloon cells, and surface erosion. 

## Conclusion

Finally, we concluded that the number of eosinophils would be undoubtedly higher in the lamina propria than other layers in all groups and the highest mean eosinophil count is indeed in the GERD group. Nonetheless, a consensus on the eosinophil count limit requires more comprehensive studies using adequate sample size designed in the matched clinical and pathological categories of different geographical regions.

## Conflict of Interest

The authors declare no conflict of interest.

## Funding

This article is an independent study that was conducted without organizational financial support.
